# Interpretation threshold values for patient-reported outcomes in patients participating in a digitally delivered first-line treatment program for hip or knee osteoarthritis

**DOI:** 10.1016/j.ocarto.2023.100375

**Published:** 2023-05-20

**Authors:** Anna Cronström, Lina H. Ingelsrud, Håkan Nero, L Stefan Lohmander, Majda Misini Ignjatovic, Leif E. Dahlberg, Ali Kiadaliri

**Affiliations:** a Department of Health Sciences, Lund University, Sweden; b Department of Community Medicine and Rehabilitation, Umeå University, Sweden; c Department of Orthopaedic Surgery, Copenhagen University Hospital Hvidovre, Denmark; d Department of Clinical Sciences Lund, Orthopedics, Lund University, Sweden; e Department of Clinical Sciences Lund, Orthopedics, Lund University, Arthro Therapeutics AB, Malmö, Sweden; f Arthro Therapeutics AB, Malmö, Sweden; g Department of Clinical Sciences Lund, Clinical Epidemiology Unit, Orthopedics, Lund University, Arthro Therapeutics AB, Malmö, Sweden

**Keywords:** Osteoarthritis, Minimal important change, Patient acceptable symptom state, Treatment failure

## Abstract

**Objective:**

Establish proportions of patients reporting important improvement, acceptable symptoms and treatment failure and define interpretation threshold values for pain, patient-reported function and quality-of-life after participating in digital first-line treatment including education and exercise for hip and knee osteoarthritis (OA).

**Methods:**

Observational study. Responses to the pain Numeric Rating Scale (NRS, 0–10 best to worst), Knee injury and Osteoarthritis Outcome Score 12 (KOOS-12) and Hip disability and Osteoarthritis Outcome Score 12 (HOOS-12, both 0–100 worst to best) were obtained for 4383 (2987) and 2041 (1264) participants with knee (hip) OA at 3 and 12 months post intervention. Threshold values for Minimal Important Change (MIC), Patient Acceptable Symptom State (PASS) and Treatment Failure (TF) were estimated using anchor-based predictive modeling.

**Results:**

70–85% reported an important improvement in pain, function and quality of life after 3 and 12 months follow-up. 42% (3 months) and 51% (12 months) considered their current state as satisfactory, whereas 2–4% considered treatment failed. MIC values were −1 (NRS) and 0–4 (KOOS/HOOS-12) across follow-ups and joint affected. PASS threshold value for NRS was 3, and 53–73 for the KOOS/HOOS-12 subscales Corresponding values for TF were 5 (NRS) and 34–55 (KOOS/HOOS-12). Patients with more severe pain at baseline had higher MIC scores and accepted poorer outcomes at follow-ups.

**Conclusion:**

Threshold estimates aid in the interpretation of outcomes after first-line OA interventions assessed with NRS Pain and KOOS/HOOS-12. Baseline pain severity is important to consider when interpreting threshold values after first-line interventions in these patients.

## 1 Background

Patient-reported outcome measures (PROMs) are recommended as the primary end-point in clinical trials evaluating treatment effects in medical conditions [1]. For a meaningful interpretation of the PROMs used, different approaches have been proposed [2], such as the Minimal Important Change (MIC), which is the smallest change in scores that represents an important improvement for the average patient, i.e., the patient is feeling better [3], the Patient Acceptable Symptom State (PASS), which represents the cut-off for the health status that the average patient considers acceptable, i.e., the patient is feeling good [4,5], and Treatment Failure (TF), which represents the cut-off below which the average patient considers their state so unsatisfactory that they think the treatment has failed [2], all based on relevant anchor questions.

Previous research in patients with anterior cruciate ligament (ACL) injury [2] and femoroacetabular impingement [6] revealed that feeling better is not necessarily the same as feeling good and reporting only the MIC (or mean change) may overestimate the results. For the patient, reaching an acceptable state of symptoms seems to be more important than feeling better [5], stressing the need for including more than a single measure of improvement to evaluate treatment effects on PROMs.

In patients with hip and/or knee osteoarthritis (OA), the Numeric Rating Scale (NRS), evaluating joint pain, and the Knee Injury and Osteoarthritis Outcome Score (KOOS) or the Hip disability and Osteoarthritis Outcome Score (HOOS), evaluating joint pain, symptoms, function and health-related quality of life are commonly used PROMs for assessing improvement during rehabilitation/treatment [7]. Although thresholds for MIC, PASS and TF have been established for the KOOS in patients after ACL injury [2,8,9] and meniscal surgery [10], such thresholds are not clearly defined in patients with OA. MIC values ranging between −1.5 and 21 were reported for the different KOOS subscales at 1-year follow-up after non-surgical treatment of knee OA [11], while an MIC between 12 and 15 points for the KOOS subscales was found after 4 weeks of physical therapy treatment for knee OA [12]. However, the anchor questions used in these studies seem not to have been domain based, which may have violated the validity of the results [13]. A few studies have examined the MIC of a subset of subscales (pain, quality of life) or short-forms of the KOOS/HOOS (PS) scores after surgical procedures, such as total knee replacement or hip arthroplasty [[14], [15], [16]]. The specific cut-offs that are relevant for patients are suggested to be population and context-based [17] and the MIC, PASS and TF for the KOOS/HOOS and NRS in knee and hip OA patients undergoing first-line treatment, including education and exercise, for hip or knee OA remain to be determined.

The aims of the present study were to 1) establish the proportion of patients reporting important improvement, acceptable symptom-levels and/or treatment failure at 3- and 12-month follow-ups after participating in digital first-line treatment for hip or knee OA, and 2) define MIC, PASS and TF cut-offs in HOOS/KOOS and the NRS-scales at both follow-ups, using anchor-based methodology and predictive modeling.

## 2 Methods

This was a retrospective analysis of prospectively collected data, adhering to the STROBE-guidelines for observational studies [18]. The study was approved by the Swedish Ethical Review Board (2021-06-16, Dnr 2021-01713) and pre-registered at ClinicalTrials.gov (NCT05316194). All participants gave digital consent prior to data extraction and analysis for research.

### 2.1 Intervention

All participants participated in a digitally delivered first-line treatment program for hip or knee OA, described in detail [19]. The program is app-based, inspired by the Swedish face-to-face management program for OA “Better management of patients with OsteoArthritis” [20] and includes weekly educational sessions, individualized exercises and a possibility to chat asynchronously with a physical therapist during the entire duration of the program. Data for participant demographics (e.g., sex, age, painful joint (hip/knee), body mass index (BMI), educational level) were collected at registration. All participants were asked to answer a set of PROMs at the beginning of treatment (baseline) and at follow-ups of 3 and 12 months as well as anchor questions at both follow-ups.

### 2.2 Participants

Data for all participants enrolled in the program from inception to July 2021 were extracted from the digital treatment register. Inclusion criteria were i) diagnosed hip or knee OA, ii) registered to the digital program and having at least one session with the physical therapist and two weeks of program participation, and iii) provided answer for KOOS/HOOS questionnaires and/or NRS pain at baseline and any of the follow-ups at 3 (10–14 weeks) and/or 12 months (50–54 weeks), and to the anchor questions at any follow-up.

### 2.3 Questionnaires and anchor-based questions

The short versions of the KOOS (KOOS-12) (knee OA) and HOOS (HOOS-12) (hip OA) [21,22] were used for evaluating knee/hip pain, function and quality of life. KOOS/HOOS-12 [23] include 12 items of the original KOOS/HOOS questionnaires [23] measuring knee/hip pain, physical function and knee/hip-related quality of life. All items were scored from 0 to 4. The scores were then normalized to a score from 0 to 100 for each domain (pain, function and quality of life) as well as a total score of all three domains where 0 indicated extreme problems and 100 indicated no problems.

Joint pain was measured with the NRS-scale. NRS comprises an 11-point scale where 0 indicated no pain and 10 indicated the worst possible pain during the last week [24].

The MIC, PASS and TF associated with KOOS/HOOS-12 (for each domain and for total score) and NRS scores, respectively, were calculated using domain specific anchor questions adapted from previous studies [8,9], all outlined in Table 1. For the total KOOS/HOOS-12 score, the anchor question with the highest correlation to the total score was used. For the MIC score, participants had 7 response options ranging from “an important improvement” to “an important deterioration”, whereas the PASS and TF questions had a dichotomous reply (yes/no) (Table 1).

**Table 1:**
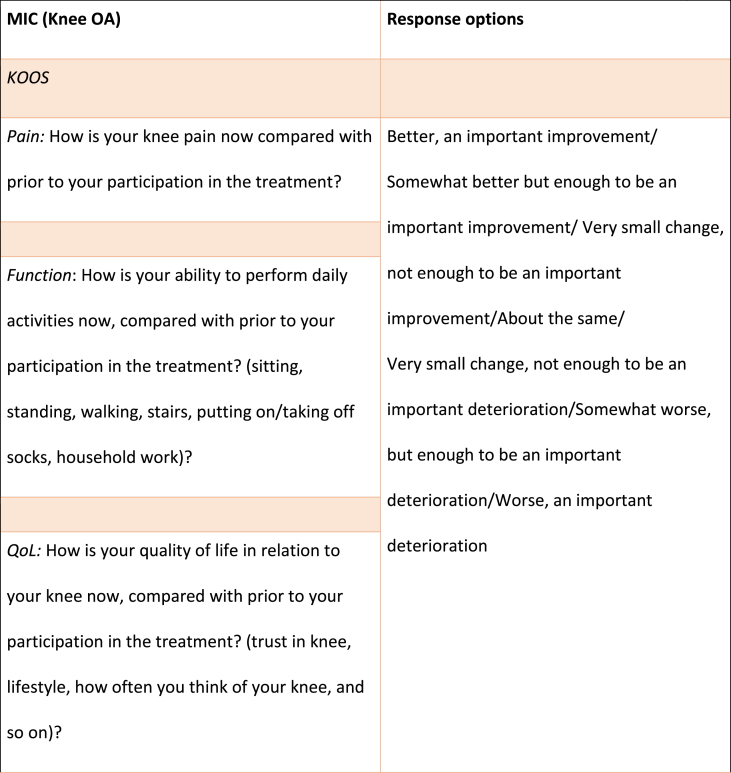

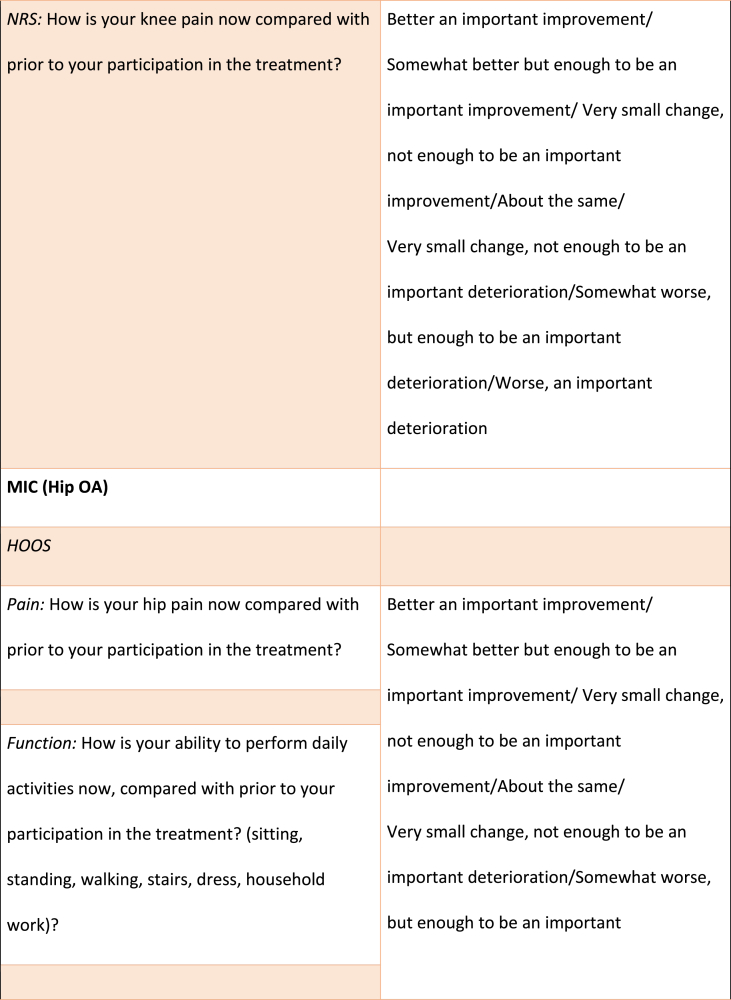

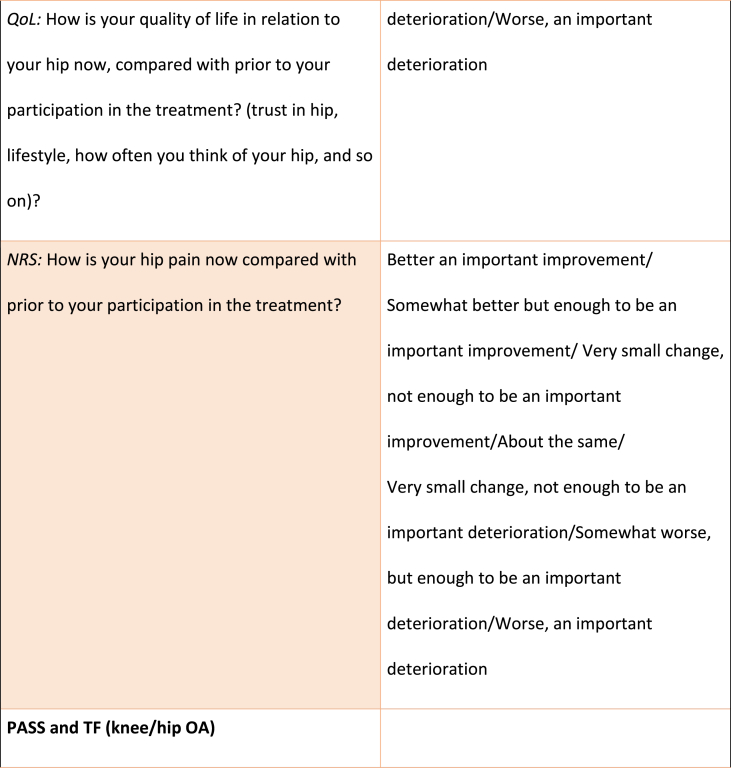

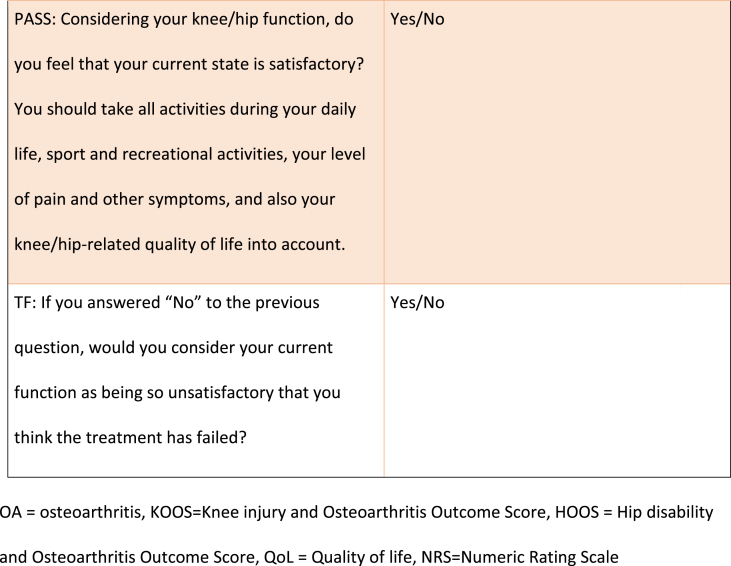
Anchor-questions used to evaluate Minimal Important Change (MIC), Patient Acceptable Symptom State (PASS) and Treatment Failure (TF).

### 2.4 Statistical analysis

For MIC calculations, anchor-responses were graded as “importantly improved” if they answered “Better, an important improvement” or “Somewhat better but enough to be an important improvement”. MIC for an important improvement was then calculated with the predictive modeling method using logistic regression analysis with improved/not improved as dependent variable and the specific PROM change score as independent variable. This method provides more precise estimates than the more traditional method based on receiver operating characteristic analysis [25]. Additionally, predictive modeling allows to adjust for the bias that results from having uneven distributed proportion reporting being importantly improved [26]. The MIC value corresponds to the change score that is equally likely in respondents reporting improvement and in those reporting no improvement and reflects the average unmeasurable individual thresholds for important improvement [25,26].

The predictive modeling method was also used to calculate the post score (i.e., 3 and 12 months) for KOOS/HOOS-scores and NRS-scores for participants that had responded “Yes” to the PASS or TF anchor question, respectively. The proportion of participants reaching MIC, PASS and TF for the KOOS/HOOS and NRS based on the response to the anchor questions, were calculated separately for participants with knee and hip OA.

Subgroup analyses on the effect of baseline severity on MIC, PASS and TF were also performed using the predictive modeling method [25,26]. To avoid potential bias associated with baseline dependency calculations, all participants were divided into two groups (high/low severity) using baseline mean NRS pain scores for KOOS/HOOS analyses and mean KOOS/HOOS pain scores for the NRS analyses as cut-off, in line with previous description [27]. Sensitivity analyses for MIC scores including only participants with responses at both 3 and 12 months follow-up were calculated using the predictive modeling method.

## 3 Results

Data for 11,708 participants were extracted from the registry whereof 6952 and 4756 had knee and hip OA, respectively. Of these, 2058 (29.6%) of participants with knee OA and 1507 (31.7%) of those with hip OA had no follow-up response and were excluded. Of the remaining, we included 4383 (63% of total sample) participants with knee OA at 3 months follow-up and 2041 (29% of total sample) at 12-month follow-up with complete data on the PROMs and anchor questions. Corresponding numbers for hip OA were 2987 (63% of total sample) and 1264 (27% of total sample) individuals (See Table 2 for characteristics).

**Table 2:** Baseline characteristics of included and excluded participants.

	Included (3-month and/or 12-month)		Excluded	
	Knee (n ​= ​4894)	Hip (n ​= ​3249)	Knee (n ​= ​2058)	Hip (n ​= ​1507)
Female, n (%)	3625 (74.1)	2532 (77.9)	1544 (75.0)	1175 (78.0)
Age, mean (SD)	65.0 (8.8)	65.0 (8.9)	62.6 (9.6)	63.2 (9.9)
24–50 years, n (%)	277 (5.7)	183 (5.6)	225 (10.9)	151 (10.0)
51–65 years, n (%)	2164 (44.2)	1413 (43.5)	1028 (50.0)	737 (48.9)
66–74 years, n (%)	1786 (36.5)	1212 (37.3)	577 (28.0)	429 (28.5)
75+ years, n (%)	667 (13.6)	441 (13.6)	228 (11.1)	190 (12.6)
Education, n (%)				
Less than high school	440 (9.0)	312 (9.6)	141 (6.9)	122 (8.1)
High school	1746 (35.7)	1163 (35.8)	722 (35.1)	498 (33.1)
College/university	2708 (55.3)	1774 (54.6)	1195 (58.1)	886 (58.8)
Body mass index, mean (SD)	27.3 (4.8)	26.3 (4.3)	28.0 (5.1)	27.1 (5.2)
Employment, n (%)				
Working	1954 (39.9)	1284 (39.5)	1055 (51.3)	708 (47.0)
Not working	244 (5.0)	142 (4.4)	125 (6.1)	97 (6.4)
Retired	2696 (55.1)	1823 (56.1)	878 (42.7)	702 (46.6)
Pain, mean (SD)	5.1 (2.0)	5.1 (1.9)	5.1 (2.0)	5.1 (2.0)
Physical function, mean (SD)	12.6 (4.2)	12.9 (4.4)	12.8 (4.5)	13.0 (4.6)
KOOS-12, mean (SD)				
Pain	52.7 (16.7)	–	53.3 (16.5)	
Function	61.7 (19.2)	–	62.2 (19.0)	
Quality of life	43.9 (16.6)	–	44.2 (17.0)	
Total	52.8 (15.4)	–	53.2 (15.3)	
HOOS-12, mean (SD)				
Pain	–	52.6 (15.7)		52.1 (16.9)
Function	–	63.5 (18.9)		63.2 (19.5)
Quality of life	–	48.8 (17.1)		47.8 (17.9)
Total	–	55.0 (15.1)		54.4 (16.1)
Follow up participation, n (%)				
Only 3-month	2858 (58.4)	1992 (61.3)		
Only 12-month	516 (10.5)	282 (8.7)		
Both 3- and 12-month	1520 (31.1)	975 (30.0)		

SD ​= ​standard deviation, KOOS = Knee injury and Osteoarthritis Outcome Score, HOOS = Hip disability and Osteoarthritis Outcome Score (KOOS/HOOS-12 both 0–100 worst to best).

### 3.1 MIC

The correlations between the KOOS/HOOS and NRS scores and the anchor questions ranged between −0.34 and −0.52 (Appendix A, Table 1). The anchor with the highest correlation to KOOS/HOOS total was the question regarding pain and this anchor was used in the analyses for total scores.

Proportions of participants importantly improved at 3 and 12 months ranged from 69.5 to 84.3% for the different KOOS/HOOS and NRS scores ().

For participants with knee OA, the MIC for improvement at 3 months was −1 for NRS and ranged between 2 and 4 for the different KOOS subscales and total score. The corresponding values at 12 months were −1 (NRS) and 0–4 (KOOS subscales and total score) ().

**Fig. 2:**
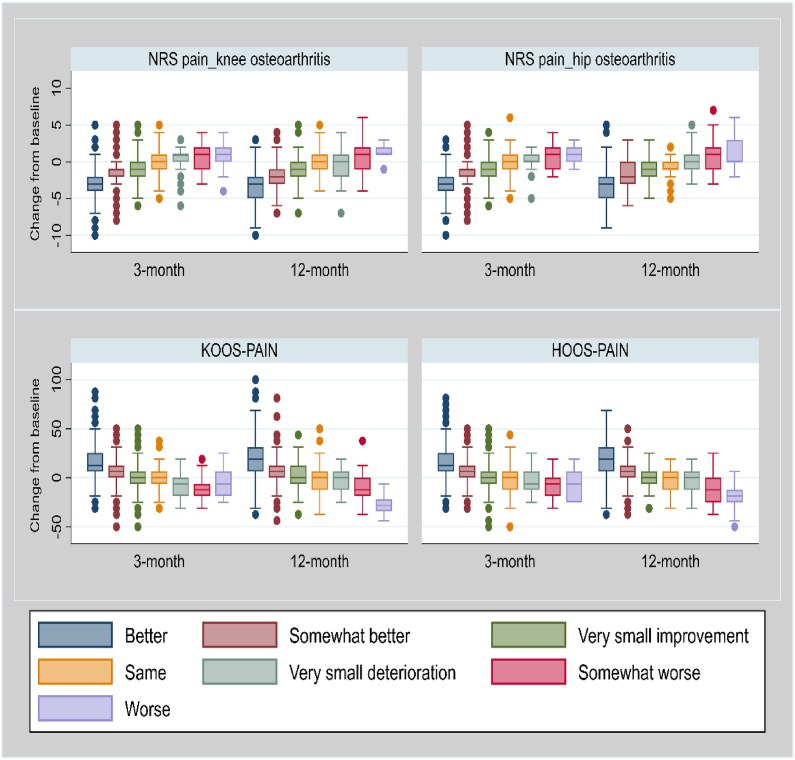
Changes in NRS pain and KOOS/HOOS-12 pain scores by anchor question response categories, stratified by follow up month (KOOS/HOOS-12 both 0–100 **worst** to best). The box includes the first to the third quartile, the median value is indicated by the black line through the box, the circles indicate outliers and the error bars represents extreme values within 1.5 interquartile values.

**Fig. 3:**
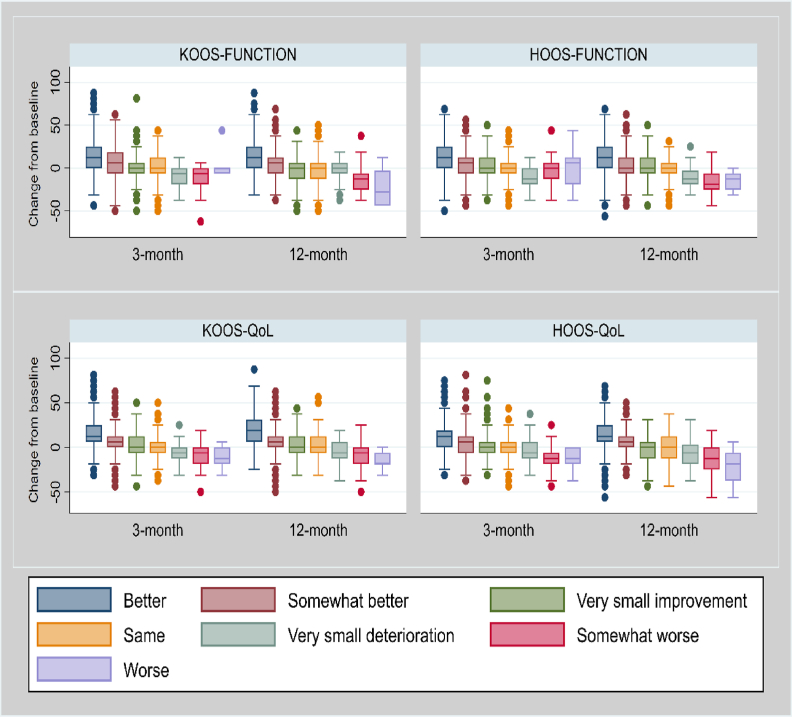
**Changes** in KOOS/HOOS-12 function and quality of life scores by anchor questions response categories, stratified by follow-up month (KOOS/HOOS-12 both 0–100 worst to best). The box includes the first to the third quartile, the median value is indicated by the black line through the box, the circles indicate outliers and the error bars represents extreme values within 1.5 interquartile values.

**Fig. 4:**
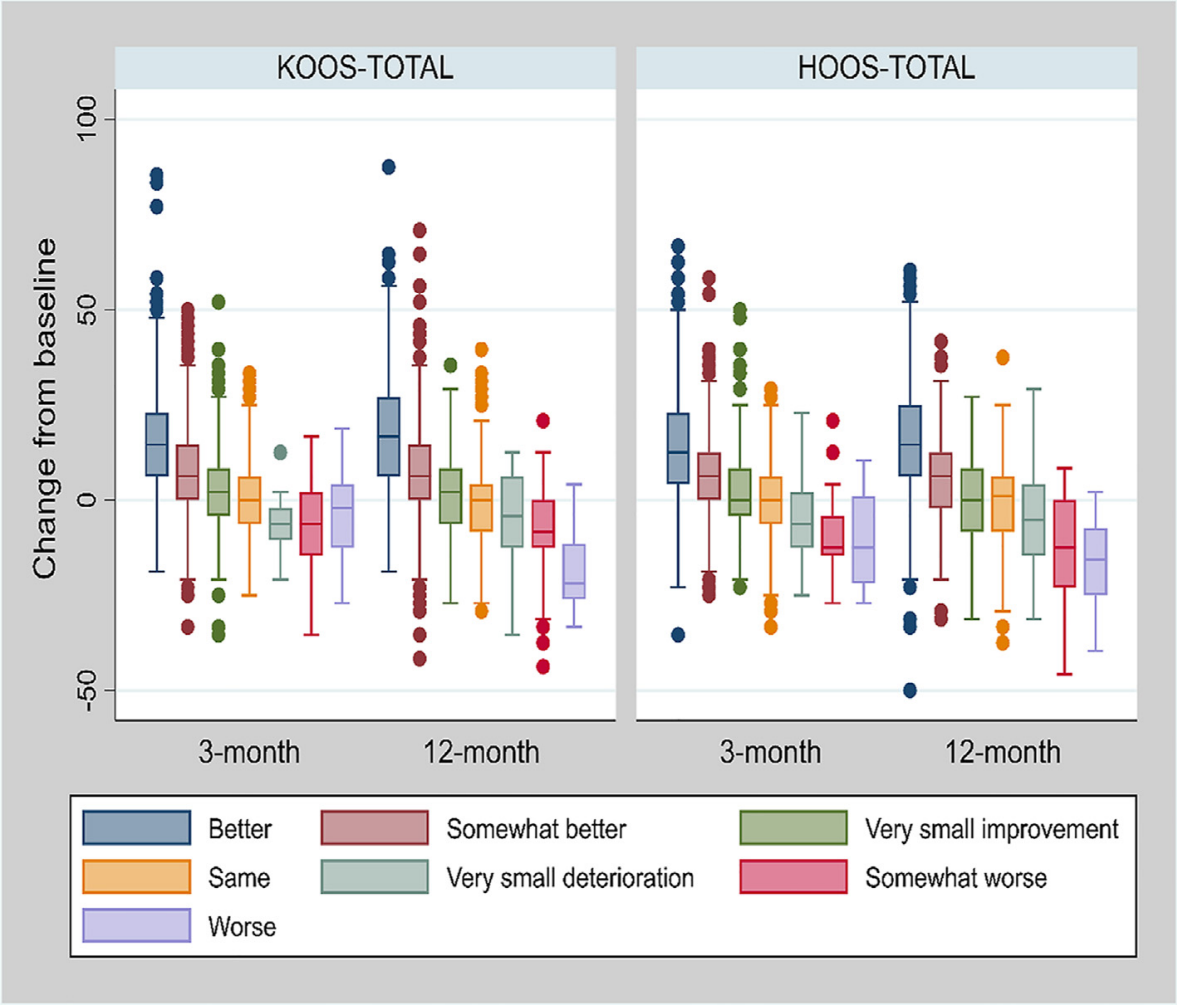
**Changes** in KOOS/HOOS-12 total scores by anchor questions response categories, stratified by follow-up month (KOOS/HOOS-12 both 0–100 worst to best). The box includes the first to the third quartile, the median value is indicated by the black line through the box, the circles indicate outliers and the error bars represents extreme values within 1.5 interquartile values.

For participants with hip OA, the MIC for improvement at 3 months was −1 for NRS and ranged between 2 and 3 for the different HOOS subscales and total score. The corresponding values at 12 months were −1 (NRS) and 0–4 (HOOS subscales and total score) (Table 4 and Appendix B, Figs. 2–4).

**Table 3:** Proportions of participants that reported an important improvement based on the anchor questions for pain, function, quality of life, acceptable symptom state and treatment failure at 3 and 12 months.

	Knee OA n (%)	Hip OA n (%)
Pain		
3 ​m	3490 (79.7)	2289 (77.2)
12 ​m	1716 (84.3)	1030 (81.9)
Function		
3 ​m	3235 (73.9)	2073 (69.9)
12 ​m	1632 (80.2)	973 (77.4)
Quality of life		
3 ​m	3041 (69.5)	2045 (68.9)
12 ​m	1577 (76.5)	951 (75.7)
PASS		
3 ​m	1869 (42.6)	1258 (42.1)
12 ​m	1045 (51.2)	658 (51.3)
TF		
3 ​m	150 (3.4)	118 (4.0)
12 ​m	49 (2.4)	21 (2.1)

OA ​= ​osteoarthritis, m ​= ​months, PASS = Patient acceptable Symptom State, TF ​= ​Treatment Failure.

**Table 4:** Minimal Important Change values for KOOS-12/HOOS-12 and NRS scores at 3 and 12 months follow-up using the predictive modeling method.

	Adjusted predictive modeling scores (95% CI)			
	Knee OA		Hip OA	
	3 months (n ​= ​4378)	12 months (n ​= ​2036)	3 months (n ​= ​2987)	12 months (n ​= ​1264)
NRS pain	−1.0 (−0.9; −1.1)	−1.1 (−1.0; −1.3)	0.9 (−0.9; −1.0)	1.0 (−0.9; −1.2)
KOOS-12/HOOS-12				
Pain subscale	2.8 (2.2; 3.3)	1.5 (0.4; 2.5)	2.2 (1.5; 2.9)	0.7 (−0.4; 1.8)
Function subscale	3.0 (2.5; 3.6)	0.4 (−0.7; 1.5)	3.0 (2.4; 3.7)	−0.1 (−1.3; 1.0)
QoL subscale	3.6 (3.1; 4.0)	3.5 (2.7; 4.3)	1.7 (1.1; 2.2)	3.5 (−0.7; 1.6)
Total KOOS/HOOS	2.3 (1.9; 2.7)	0.8 (0.0; 1.7)	1.6 (1.1; 2.1)	−0.3 (−1.3; 0.6)

OA ​= ​osteoarthritis, NRS = Numeric Rating Scale, KOOS = Knee injury and Osteoarthritis Outcome Score, HOOS = Hip disability and Osteoarthritis Outcome Score (KOOS/HOOS-12 both 0–100 worst to best), QoL ​= ​Quality of Life.

### 3.2 PASS

**Fig. 5:**
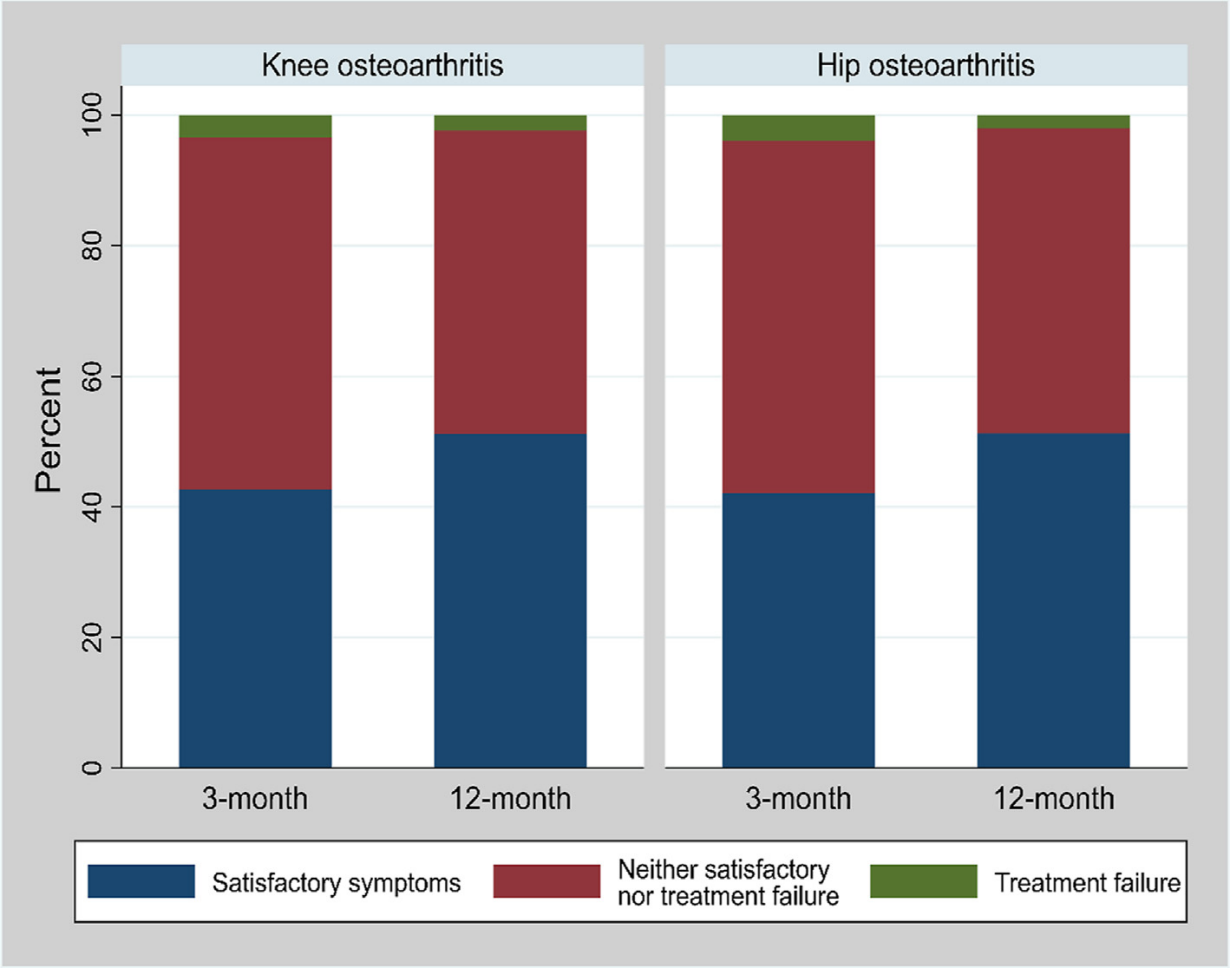
**Distributions** of responses to PASS/TF questions during follow-ups.

1869 (42.6%) participants with knee OA and 1258 (42.1%) participants with hip OA reported their current state as satisfactory at 3 months. The corresponding numbers at 12 months were 1045 (51.2%) and 658 (51.3%), respectively (Table 3 and Appendix B, Fig. 5).

NRS pain PASS thresholds at 3 and 12 months of follow-up were 3.0 and 2.7, respectively, for both knee and hip OA. KOOS/HOOS PASS (subscales and total score) thresholds at 3 and 12 months follow-up ranged between 53 and 71 (knee OA) and 56 and 73 (hip OA) (Table 5).

**Table 5:** NRS and KOOS-12/HOOS-12 scores at 3 and 12 months follow-up according to current state (PASS/TF) using the predictive modeling method.

	Adjusted predictive modeling scores (95% CI)			
	Knee OA n ​= ​4383 (3 ​m) n ​= ​2041 (6 ​m)		Hip OA n ​= ​2987 (3 ​m) n ​= ​1264 (12 ​m)	
	PASS	TF	PASS	TF
NRS pain				
3 ​m	3.0 (2.9; 3.0)	5.1 (4.9; 5.2)	3.0 (2.9; 3.1)	5.2 (5.0; 5.4)
12 ​m	2.7 (2.6; 2.8)	4.8 (4.4; 5.1)	2.7 (2.6; 2.8)	5.7 (5.2; 6.1)
KOOS/HOOS-12 pain				
3 ​m	63.4 (62.9; 63.9)	46.4 (44.9; 47.8)	62.4 (61.8; 63.0)	44.5 (43.0; 46.1)
12 ​m	63.1 (62.4; 63.8)	45.8 (43.1; 48.5)	63.1 (62.1; 64.2)	41.2 (38.1; 43.8)
KOOS/HOOS-12 function				
3 ​m	71.1 (70.5; 71.6)	54.6 (52.8; 56.4)	72.7 (72.1; 73.4)	54.1 (52.2; 56.0)
12 ​m	70.1 (69.2; 70.9)	53.2 (49.5; 56.5)	71.5 (70.5; 72.6)	50.1 (45.4; 54.5)
KOOS/HOOS-12 quality of life				
3 ​m	52.9 (52.4; 53.4)	34.5 (33.1; 36.0)	56.4 (55.8; 57.1)	35.2 (33.3; 37.0)
12 ​m	53.4 (52.8; 54.2)	33.7 (30.6; 36.5)	57.0 (56.1; 58.0)	33.9 (30.4; 37.2)
KOOS/HOOS-12 total score				
3 ​m	62.5 (62.0; 62.9)	45.7 44.4; 47.1)	63.8 (63.3; 64.4)	45.1 (43.4; 46.7)
12 ​m	62.3 (61.6; 63.0)	44.9 (42.1; 47.7)	63.9 (63.1; 64.8)	42.3 (38.9; 45.3)

OA ​= ​osteoarthritis, NRS = Numeric Rating Scale, KOOS = Knee injury and Osteoarthritis Outcome Score, HOOS = Hip disability and Osteoarthritis Outcome Score (KOOS/HOOS-12 both 0–100 worst to best).

### 3.3 TF

150 (3.4%) participants with knee OA and 118 (4.0%) participants with hip OA reported their current function so unsatisfactory that they considered that the treatment had failed at 3 months. The corresponding number of participants at 12 months were 49 (2.4%) and 21 (2.1%) (Table 3 and Appendix B, Fig. 5).

**Fig. 6:**
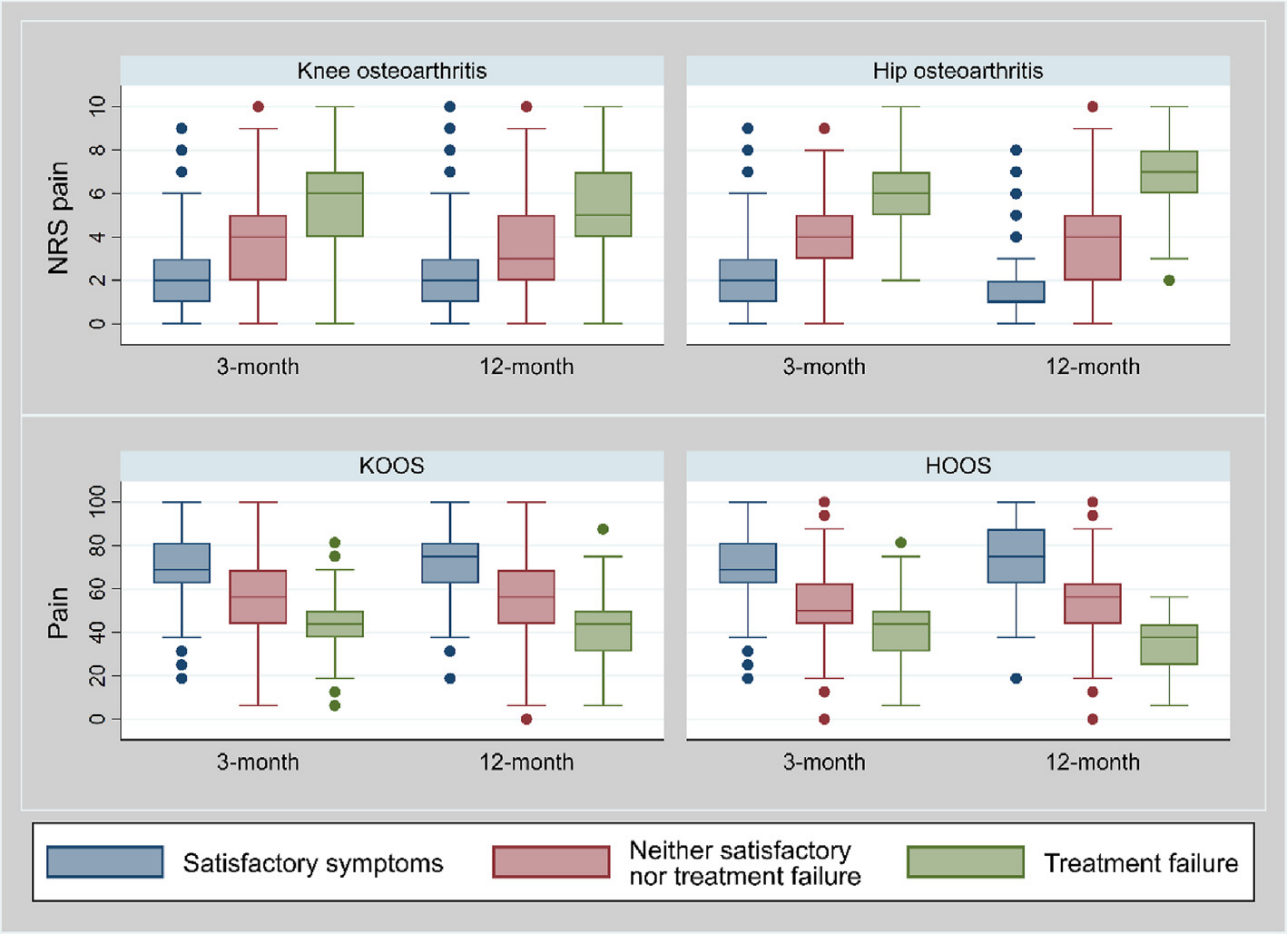
NRS **and** KOOS/HOOS-12 pain according to PASS and Treatment failure status, stratified by follow-up month (KOOS/HOOS-12 both 0–100 worst to best). The box includes the first to the third quartile, the median value is indicated by the black line through the box, the circles indicate outliers and the error bars represents extreme values within 1.5 interquartile values.

**Fig. 7:**
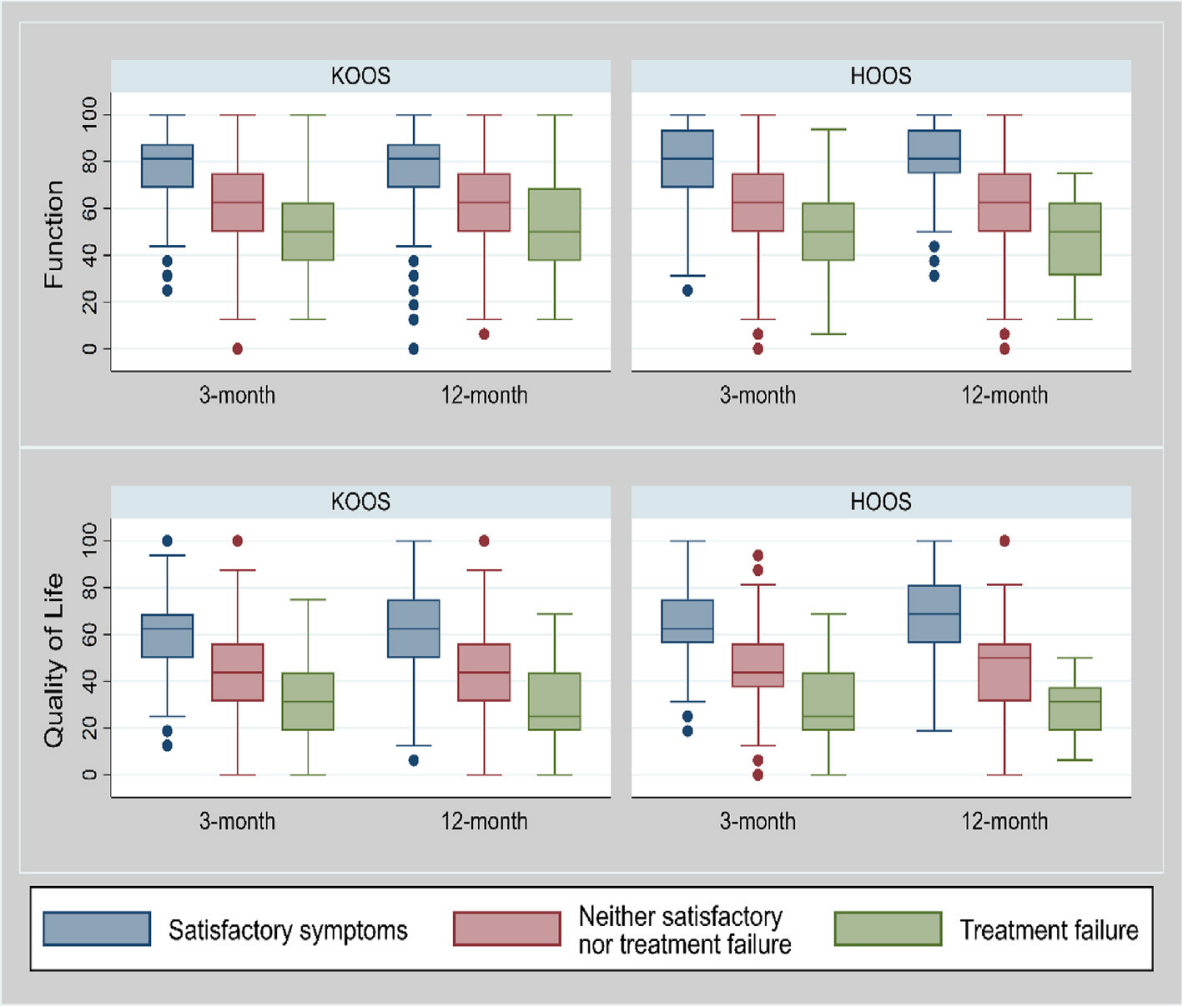
KOOS/HOOS-12 function and quality of life scores according to PASS and Treatment failure status, stratified by follow-up month (KOOS/HOOS-12 both 0–100 **worst** to best). The box includes the first to the third quartile, the median value is indicated by the black line through the box, the circles indicate outliers and the error bars represents extreme values within 1.5 interquartile values.

**Fig. 8:**
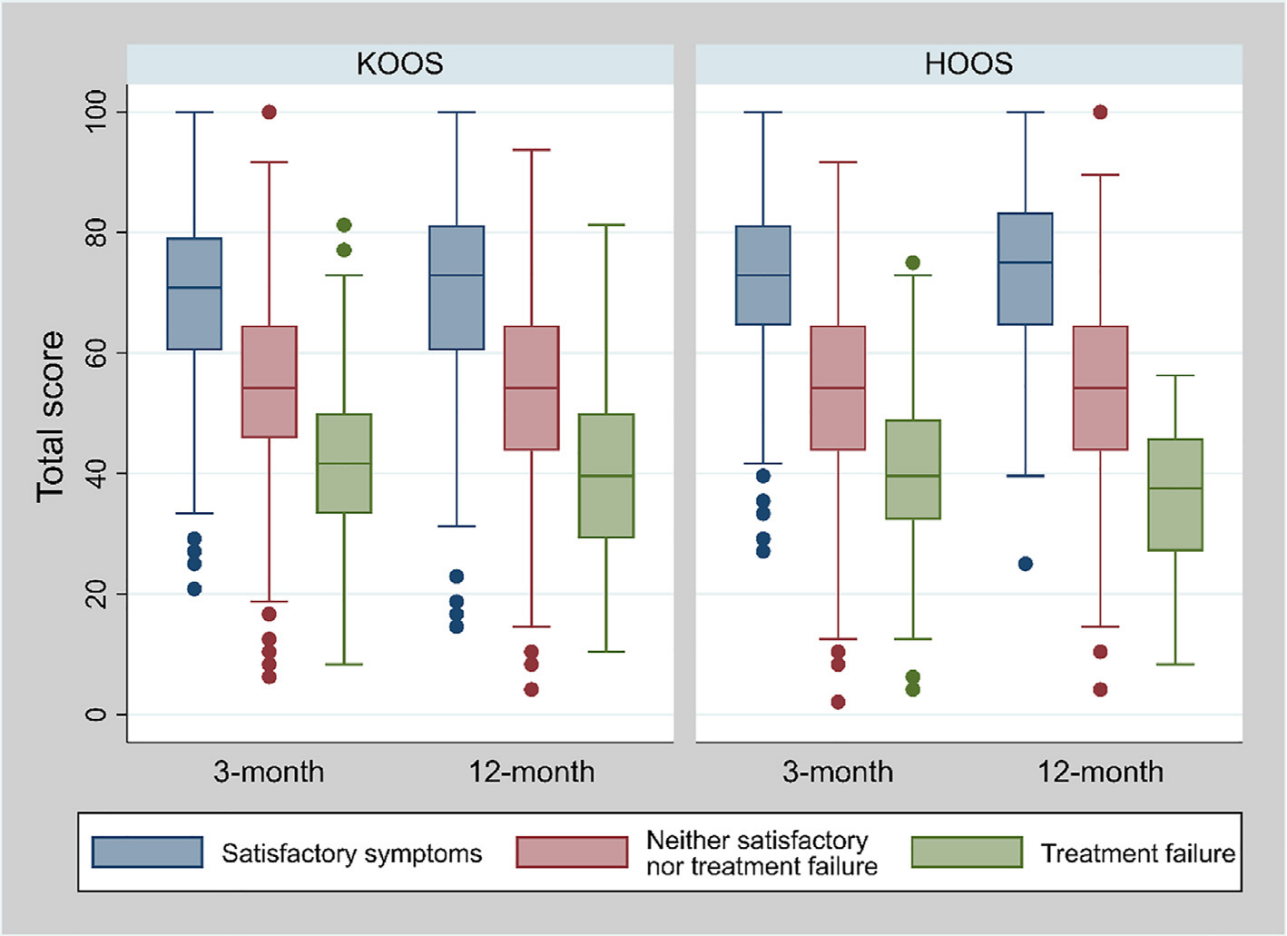
KOOS/HOOS-12 total scores according to PASS and Treatment failure status, stratified by follow-up month (KOOS/HOOS-12 both 0–100 worst to best). The **box** includes the first to the third quartile, the median value is indicated by the black line through the box, the circles indicate outliers and the error bars represents extreme values within 1.5 interquartile values.

NRS pain TF thresholds at 3 and 12 months follow-up were 5.1 and 4.8 (knee OA) and 5.2 and 5.7 (hip OA), respectively. The corresponding KOOS/HOOS TF thresholds at 3 and 12 months follow-up ranged between 34 and 55 (knee OA) and 34 and 54 (hip OA) (Table 5 and Appendix B, Figs. 6–8).

### 3.4 Subgroup analysis

The subgroup analysis on the effect of baseline pain severity showed that the MIC values were highly dependent on baseline pain severity. Thus, in patients with worse symptoms at baseline, MIC thresholds reflected larger needed absolute improvement in comparison to patients with lesser symptom levels, while PASS and TF thresholds in those with worse symptoms at baseline reflected poorer outcomes, i.e. those with greater baseline symptom severity accepted a poorer health state as satisfactory. This was true for both pain and KOOS/HOOS-12 scores (subscales and total) irrespective of joint affected (Appendix A, Tables 2 and 3).

### 3.5 Sensitivity analysis

The analysis of NRS pain MIC values at 3 and 12 months, including only those responding at both follow-ups, indicated no important change in the results (Appendix A, Table 4).

## 4 Discussion

In the present study, at least 70% of the participants reported an important improvement in pain, function and quality of life after 3–12 months participation in a digital first-line treatment for hip and knee OA. Forty-two and 51% considered their current state as satisfactory at 3 and 12 months, respectively, whereas only a few (2–4%) considered that the treatment had failed, irrespective of joint affected. One unit improvement in pain on the NRS scale was considered an MIC at both follow-ups for both patients with knee OA and hip OA. The corresponding values for the different KOOS/HOOS-12 subscales ranged from 0 to 4. Participants with worse symptoms at baseline required larger improvements to be considered an MIC for all PROMs at follow-up and accepted poorer outcomes in relation to PASS and TF compared to those with lesser baseline symptoms, indicating that all interpretation threshold values were highly dependent on baseline severity.

We found a one unit reduction on the NRS to represent MIC at both 3 and 12 months follow-up after participation in a digital first-line treatment for hip and knee OA. This result establishes this cut-off as an MIC in pain in people with hip and knee OA and could thus be used as a clinically relevant cut-off in future research trials in this population.

The MIC scores reported for the different KOOS/HOOS-12 subscales in this study ranged between 0 and 4 for both follow-ups. This is a considerably lower MIC than most of the scores previously reported in patients with knee OA after non-surgical treatment, where MICs between 3 and 18 [11] and 12 to 15 [12] were observed for the different subscales of the full KOOS questionnaire. There may be several reasons for this discrepancy. In contrast to the studies by Mills et al. [11] and Mostafaee et al. [12], we used the short versions (KOOS/HOOS-12) [21,22] of the original KOOS/HOOS questionnaires as well as specific domain based anchor questions for each subscale instead of the same anchor across subscales. In addition, we used the predictive modeling method instead of ROC-analysis since recent research proposed the predictive modeling approach to be a more precise measure when evaluating these thresholds [25]. Unlike ROC-analysis, the predictive modeling method takes into account unequal variances between groups and prevalence of improved patients [25,26], which may be a reason for the lower values in our study.

Mahler et al. reported a PASS cut-off of 53 for KOOS-physical function (PS) after 3 months participation in a *stepped care approach* including education, physical therapy and analgesics for knee OA [28]. The KOOS-PS was derived from the two KOOS subscales Activity of Daily Living and Sport recreation and holds a different set of items than the KOOS/HOOS-12 function subscales used in the current study. Mahler et al. used a generic anchor (i.e., not specifically related to function) and the PASS score was calculated using the 75th percentile of the cumulative KOOS-PS score, which may further explain the differences between that study and the current study. The PASS cut-offs for KOOS/HOOS-12 scores (subscales; pain, function and quality-of-life) ranged between 53 and 73 in the current study, further highlighting that no single PASS cut-off should be used across KOOS/HOOS dimensions.

Although approximately 70%–80% reported to have experienced an important improvement in all PROMs after program participation, similar to the study by Mahler et al. [28], only around 40%–50% reported an acceptable symptom state at the different follow-ups. Experiencing an important improvement is, as reported in e.g. patients with ACL injury [2], not the same as being satisfied with the current state of function or symptoms. Using the MIC as the only responder criterion may overestimate the results also in patients with hip and knee OA. The PASS score for pain was approximately 3 on the NRS scale, irrespective of time point (3 vs. 12 months) and joint affected, while the corresponding values for KOOS/HOOS-12 scores ranged between 53 and 73. The relatively small changes in PROM scores (−1 (NRS), 0–4 KOOS/HOOS)) that were considered an MIC by the patients implies that even though an important improvement was perceived, many patients did not reach the state they would consider acceptable. It is, therefore, important to include not only a single interpretation threshold measure such as MIC in rehabilitation and research to gain a fair and full picture of the results of a specific intervention.

Not all individuals will benefit from education and exercise for OA [29]. In the present study, around 2–4% considered their symptoms so severe after program participation that they thought the treatment had failed. The TF threshold for the NRS score for these patients was 5 and below 54 to 33 on the different KOOS/HOOS-12 subscales at both follow-ups. Previous studies suggest that both adherence to treatment [29] and co-morbidities, such as obesity, depression and cardiovascular disease, may be factors relevant for not responding to treatment [30]. A recent qualitative study revealed that those who did not respond to first-line treatment believed that their low adherence to the treatment was the reason for the unsuccessful outcome and that over-weight, comorbidities and psychological factors prevented them to fully participate in the exercises [31]. It may be important to identify potential non-responders to exercise and provide behavioral and motivational support in an effort to increase adherence to first-line treatment and thereby increase the chances for a satisfying outcome in these individuals [32]. Interpretation thresholds may add in identifying non-responders/responders to specific interventions, as well as being useful as reference in patient communication regarding treatment expectations and outcomes.

In line with studies reporting those with worse baseline symptoms to have higher threshold MIC scores for NRS and KOOS in patients with musculoskeletal disorders [33] and knee OA [11], we here found MICs in those with greater baseline pain to reflect larger improvements for all PROMs. On the other hand, those with more severe symptoms at baseline seemed to accept poorer health states at follow-up, reflected in their PASS and TF thresholds. This baseline severity dependency should be considered when interpreting MIC, PASS and TF after first-line treatment for hip and knee OA.

The large sample of patients that all participated in the same first-line treatment program for hip and knee OA is a strength of this study. Some limitations are associated with this study. To provide a valid assessment of the PROMs, the anchor questions used need to correlate with the difference in PROMs between baseline and follow-up [34]. Although the correlations between the anchor questions and the different PROMs were within the recommended acceptable range of 0.30–0.35 [34], many of the correlations were at the lower end which may have affected the result. In the baseline severity subgroup analysis, many of the MIC scores, especially for the 12 months follow-up, were negative for the low severity group, i.e., a deterioration was regarded as an important improvement. This phenomenon was reported [11], and may be attributed to recall bias and/or to a so called “response shift”, i.e., the individual may recalibrate, reprioritize or redefine the construct to be assessed over time [35,36]. Patients participating in digital treatment for OA have been reported to be younger, more often female, having higher levels of education and more often still be working, compared to those participating in traditional face-to-face version of the corresponding OA treatment [37]. Approximately 30% of the participants did not complete any of the follows-up and were excluded. While the baseline PROMs were comparable, those excluded were somewhat younger, having somewhat higher educational level and were working to a greater extent than those completing the follow-ups (Table 2). This, together with the lower response rate at 12 months follow-up may limit the generalization of interpretation thresholds reported in this study to the OA population as a whole. The sensitivity analysis including only those who responded to both follow-ups revealed no important changes to the MIC values at 3 or 12 month, indicating that the drop out rate at 12 months had minor influence of the results.

## 5 Conclusion

These interpretation threshold estimates improve our understanding and interpretation of outcomes after first-line OA interventions when assessed with the NRS Pain, KOOS-12 and HOOS-12. The baseline pain dependency of threshold estimates identified is important to consider when these values are used to interpret changes in PROMs after first-line interventions in patients with hip and knee OA.

## Author contributions

AC contributed to the conception and design of the study, was in charge of manuscript writing and contributed to the interpretation of data analysis. LHI, HN, LSL, MMI and LED contributed to the conception and design of the study, interpretation of data and manuscript writing. AK contributed to the conception and design of the study, was in charge of data analysis and contributed to writing the manuscript. All authors read and approved the final version of the manuscript. AC (anna.cronstrom@med.lu.se) and AK (ali.kiadaliri@med.lu.se) take full responsibility for the integrity of the work as a whole, from inception to finished article.

## Role of the funding source

This work was funded by Greta and Johan Kocks foundation and Stiftelsen för bistånd åt rörelsehindrade i Skåne.

## Declaration of competing interest

AC and LHI report no conflicts of interests related to the content of this manuscript. LSL was a scientific consultant for Arthro Therapeutics AB (the company providing the digital program analyzed in this study). HN and MMI were employed by Arthro Therapeutics AB during 2021–2022. LED is co-founder and chief medical officer at Arthro Therapeutics AB. AK acts as a scientific advisor (7.5% FTE) for Arthro Therapeutics AB.

## Figures and Tables

**Table 1:** Spearman correlations between the patient-reported outcome measure (PROM) change score and the anchor for each PROM presented in Table 1 (main manuscript).

	Knee OA	Hip OA
NRS pain		
3 ​m	0.52	0.52
12 ​m	0.46	0.52
KOOS/HOOS-12 pain		
3 ​m	−0.44	−0.44
12 ​m	−0.45	−0.49
KOOS/HOOS-12 function		
3 ​m	−0.32	−0.33
12 ​m	−0.30	−0.40
KOOS/HOOS- quality of life		
3 ​m	−0.34	−0.34
12 ​m	−0.44	−0.44
KOOS/HOOS-12 total score		
3 ​m	−0.47	−0.46
12 ​m	−0.50	−0.52

OA = Osteoarthritis, NRS = Numeric Rating Scale, KOOS = Knee injury and Osteoarthritis Outcome Score, HOOS = Hip disability and Osteoarthritis Outcome Score (KOOS/HOOS-12 both 0–100 worst to best).

**Table 2:** Minimal Important Change values for KOOS/HOOS and NRS scores at 3 and 12 months follow-up by baseline severity using the predictive modeling method.

	Adjusted predictive modeling scores (95% CI)											
	Knee OA						Hip OA					
	3 months			12 months			3 months			12 months		
	High severity	Low severity	Mean diff (95% CI)	High severity	Low severity	Mean diff (95% CI)	High severity	Low severity	Mean diff (95% CI)	High severity	Low severity	Mean diff (95% CI)
NRS pain	−1.4	−0.8.	−0.7	−1.7	−0.8	−0.9	−1.3	−0.7	−0.6	−1.4	−0.6	−0.8
	(-1.3; −1.5), n ​= ​1616	(-0.7; −0.8), n ​= ​2762	(-0.5, −0.8)	(-1.4; −1.9), n ​= ​745	(-0.6; −0.9), n ​= ​1291	(-0.6, −1.1)	(-1.2; −1.5), n ​= ​1101	(-0.6; −0.8), n ​= ​1866	(-0.5, −0.8)	(-1.2; −1.7), n ​= ​614	(-0.4; −0.9), n ​= ​643	(-0.5, −1.1)
KOOS/HOOS-12	n ​= ​2757	n ​= ​1621		n ​= ​1273	n ​= ​763		n ​= ​1864	n ​= ​1103		n ​= ​735	n ​= ​522	
Pain	4.5	─0.2	4.7	3.3	─1.7	5.0	3.7	─0.4	4.1	3.1	─3.1	6.2
	(3.9; 5.2), n ​= ​2757	(-1.0; 0.6),	(3.6; 5.8)	(2.1; 4.7),	(-3.6; 0.2),	(2.7; 7.2)	(2.9; 4.6),	(-1.5; 0.6),	(2.9; 5.5)	(1.7; 4.5),	(-5.2; −1.1),	(3.8; 8.5)
Function	4.6	0.6	4.0	1.5	─1.3	2.8	4.1	1.2	2.9	1.5	─2.3	3.8
	(3.8; 5.3)	(-0.2; 1.4)	(2.9; 5.0)	(0.1; 2.8)	(-2.8; 0.2)	(0.7; 4.8)	(3.2; 4.9)	(0.4; 2.1)	(1.7; 4.1)	(0.0; 3.0)	(-4.0; −0.6)	(1.7; 6.1)
QoL	4.7	1.7	3.0	4.9	1.2	3.7	2.6	0.1	2.5	1.1	─0.4	1.6
	(4.2; 5.2)	(1.0; 2.5)	(2.1; 3.8)	(3.9; 5.9)	(-0.3; 2.5)	(2.0; 5.5)	(1.9; 3.4)	(-0.7; 0.9)	(1.4; 3.6)	(-0.3; 2.6)	(-2.2; 1.3)	(-0.6; 3.9)
Total	3.7	─0.1	3.8	2.0	─1.2	3.3	2.7	─0.3	3.1	1.6	─3.4	5.0
	(3.2; 4.3)	(-0.7; 0.5)	(3.0; 4.6)	(1.0; 3.1)	(-2.8; 0.2)	(1.5; 5.0)	(2.0; 3.4)	(-1.1; 0.4)	(2.0; 4.1)	(0.3; 2.9)	(-5.2; −1.8)	(3.0; 7.1)

OA = Osteoarthritis, NRS = Numeric Rating Scale, KOOS = Knee injury and Osteoarthritis Outcome Score, HOOS = Hip disability and Osteoarthritis Outcome Score(KOOS/HOOS-12 both 0–100 worst to best), QoL ​= ​Quality of Life**.** High/low severity was calculated using baseline mean NRS pain scores for KOOS/HOOS analyses and mean KOOS/HOOS pain scores for the NRS analyses as cut-off.

**Table 3:** NRS and KOOS/HOOS-12 scores at 3 and 12 months follow-up according to current state (PASS/TF) and baseline severity using the predictive modeling method

	Adjusted predictive modeling scores (95% CI)											
	Knee OA						Hip OA					
	PASS			TF			PASS			TF		
	High severity	Low severity	Mean diff (95% CI)	High severity	Low severity	Mean diff (95% CI)	High severity	Low severity	Mean diff (95% CI)	High severity	Low severity	Mean diff (95% CI)
NRS pain												
3 ​m	3.7 (3.6; 3.8), n ​= ​1618	2.6 (2.5; 2.7), n ​= ​2765	1.1 (0.9; 1.2)	5.7 (5.4; 5.9) n ​= ​1618	4.5 (4.2; 4.7) n ​= ​2765	1.2 (0.8; 1.5)	3.5 (3.4; 3.6), n ​= ​1106	2.7 (2.6; 2.8), n ​= ​1881	0.8 (0.6; 0.9)	5.9 (5.6; 6.2) n ​= ​1106	4.4 (4.2; 4.7) n ​= ​1881	1.5 (1.1; 1.8)
12 ​m	3.1 (3.0; 3.3), n ​= ​747	2.4 (2.3; 2.5), n ​= ​1294	0.7 (0.5; 0.9)	5.3 (4.9; 5.7) n ​= ​747	4.3 (3.8; 4.8) n ​= ​1294	1.0 (0.3; 1.7)	3.0 (2.9, 3.2), n ​= ​618	2.3 (2.2, 2.4), n ​= ​646	0.7 (0.5; 1.0)	6.1 (5.5, 6.7) n ​= ​618	5.3 (4.6, 6.2) n ​= ​646	0.8 (−0.3; 2.0)
KOOS/HOOS pain												
3 ​m	59.4 (58.8; 60.1), n ​= ​2757	69.7 (69.0; 70.3), n ​= ​1621	─10.2 (−11.2; −9.3)	42.9 (41.4; 44.4) n ​= ​2757	57.0 (54.5; 59.5) n ​= ​1621	─14.1 (−16.9; −11.3)	59.5 (58.8; 60.3), n ​= ​1864	67.5 (66.6; 68.3), n ​= ​1103	─8.0 (−9.2; −6.9)	42.2.0 (40.3; 43.9) n ​= ​1864	51.0 (46.9; 55.4) n ​= ​1103	─8.8 (−13.4; −4.1)
12 ​m	60.8 (59.8; 61.8), n ​= ​1273	67.5 (66.5; 68.6), n ​= ​763	─6.7 (−8.2; −5.2)	41.9 (39.2; 44.9) n ​= ​1273	56.5 (52.2; 60.9) n ​= ​763	─14.7 (−19.7; −9.3)	60.8 (59.5; 62.1), n ​= ​735	66.1 (64.6; 67.5), n ​= ​522	─5.3 (−7.3; −3.3)	NA	NA	NA
KOOS/HOOS function												
3 ​m	67.3 (66.6; 68.1), n ​= ​2757	77.2 (76.5; 77.9), n ​= ​1621	─9.8 (−10.9; −8.8)	50.7 (48.7; 52.6) n ​= ​2757	66.2 (62.2; 69.9) n ​= ​1621	─15.4 (−19.8; −11.2)	69.5 (68.7; 70.5), n ​= ​1864	78.3 (77.4; 79.2), n ​= ​1103	─8.8 (−10.0; −7.5)	50.8 (48.7; 52.8) n ​= ​1864	64.6 (60.1; 68.7) n ​= ​1103	─13.8 (−18.2; −8.4)
12 ​m	67.4 (66.2; 68.5), n ​= ​1273	75.3 (74.1; 76.5), n ​= ​763	─7.9 (−9.6; −6.4)	48.3 (44.1; 52.2) n ​= ​1273	66.2 (59.6; 72.4) n ​= ​763	─17.9 (−25.4; −10.2)	68.7 (67.1; 70.2), n ​= ​735	75.7 (74.1; 77.2), n ​= ​522	─7.0 (−8.9; −5.0)	NA	NA	NA
KOOS/HOOS quality of life												
3 ​m	49.6 (48.9; 50.2), n ​= ​2757	58.0 (57.3; 58.6), n ​= ​1621	─8.4 (−9.4; −7.4)	31.7 (30.1; 33.3) n ​= ​2757	42.9 (39.6; 46.2) n ​= ​1621	─11.1 (−14.7; −7.4)	53.7 (52.8; 54.4), n ​= ​1864	61.1 (60.3; 62.0), n ​= ​1103	─7.5 (−8.7; −6.4)	32.6 (30.5; 34.6) n ​= ​1864	43.1 (38.6; 46.8) n ​= ​1103	─10.6 (−14.8; −5.9)
12 ​m	51.4 (50.5; 52.3), n ​= ​1273	57.4 (56.3, 58.6), n ​= ​763	─6.1 (−7.6; −4.5)	30.3 (27.0; 33.4) n ​= ​1273	43.5 (38.6; 47.6) n ​= ​763	─13.3 (−18.5; −7.7)	54.5 (53.3; 55.8), n ​= ​735	60.4 (59.0; 61.9), n ​= ​522	─5.9 (−7.9; −4.0)	NA	NA	NA
KOOS/HOOS total score												
3 ​m	58.7 (58.1; 59.3), n ​= ​2757	68.4 (67.8; 68.9), n ​= ​1621	─9.6 (−10.5, −8.8)	42.3 (40.9; 43.7) n ​= ​2757	56.3 (53.6; 58.7) n ​= ​1621	─14.0 (−16.8; −11.0)	60.7 (60.0; 61.4), n ​= ​1864	69.0 (68.3; 69.7), n ​= ​1103	─8.3 (−9.3; −7.3)	42.3 (40.6; 43.9) n ​= ​1864	53.8 (50.1; 56.8) n ​= ​1103	─12.5 (−16.2; −8.6)
12 ​m	59.8 (59.0; 60.7), n ​= ​1273	66.9 (65.9; 67.8), n ​= ​763	─7.0 (−8.4, −5.6)	40.8 (37.9; 43.6) n ​= ​1273	56.2 (51.9; 60.6) n ​= ​763	─15.4 (−20.9; −10.2)	61.2 (60.1; 62.5), n ​= ​735	67.6 (66.3; 68.9), n ​= ​522	─6.4 (−8.3; −4.6)	NA	NA	NA

OA = Osteoarthritis, NRS = Numeric Rating Scale, KOOS = Knee injury and Osteoarthritis Outcome Score, HOOS = Hip disability and Osteoarthritis Outcome Score (KOOS/HOOS-12 both 0–100 worst to best), QoL ​= ​Quality of Life, NA ​= ​not available due to low sample size. High/low severity was calculated using baseline mean NRS pain scores for KOOS/HOOS analyses and mean KOOS/HOOS pain scores for the NRS analyses as cut-off.

**Table 4:** Minimal Important Change values for NRS and KOOS/HOOS-12 scores at 3 and 12 months follow-up using the predictive modeling method including participants with responses at both follow-ups.

	Adjusted predictive modeling scores (95% CI)			
	Knee OA		Hip OA	
	3 months (n ​= ​1520)	12 months (n ​= ​1520)	3 months (n ​= ​975)	12 months (n ​= ​975)
NRS pain	−0.9 (−0.8; −1.0)	−1.2 (−1.0; −1.3)	−0.9 (−0.8; −1.1)	−1.0 (−0.9; 1.2)
KOOS/HOOS-12				
Pain subscale	2.9 (1.9; 3.7)	1.6 (0.4, 2.8)	2.4 (1.1; 3.4)	0.1 (−1.2; 1.4)
Function subscale	1.7 (0.8; 2.6)	─ 0.3 (−1.5, 0.9)	2.8 (1.8; 3.8)	─ 0.2 (−1.5; 1.0)
QoL subscale	3.3 (2.5; 4.1)	3.2 (2.2; 4.1)	1.0 (0.0; 2.0)	─ 0.1 (−1.3; 1.2)
Total KOOS/HOOS	2.1 (1.4; 2.9)	0.4 (−0.6; 1.4)	1.5 (0.7; 2.4)	─ 0.8 (−2.0; 0.3)

OA ​= ​osteoarthritis, NRS = Numeric Rating Scale, KOOS = Knee injury and Osteoarthritis Outcome Score, HOOS = Hip disability and Osteoarthritis Outcome Score (KOOS/HOOS-12 both 0–100 worst to best), QoL ​= ​Quality of Life.

**Fig. 1:**
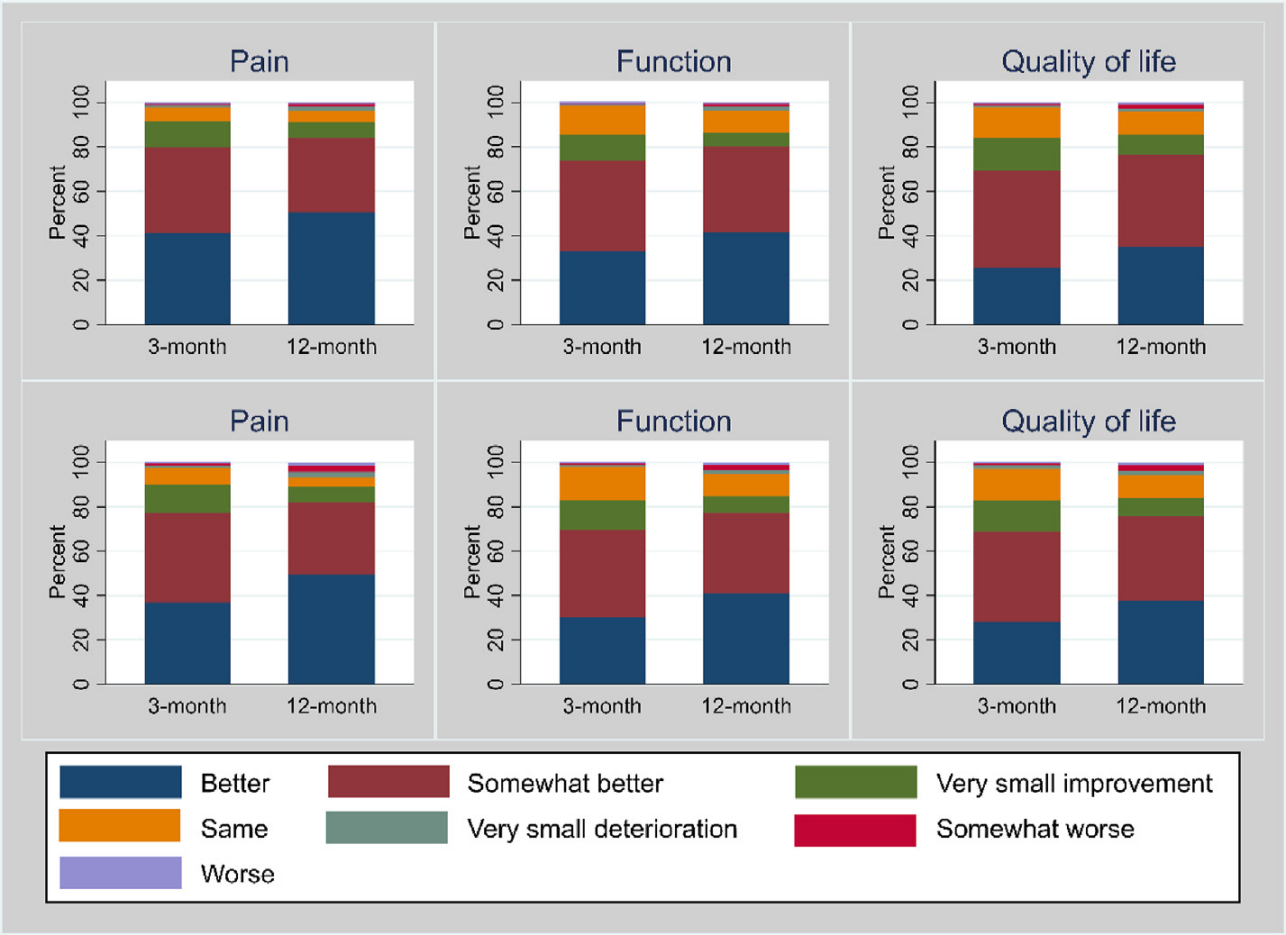
Distribution of responses to MIC anchor questions at the follow-ups for participants with knee (top) and hip (bottom) osteoarthritis.
